# Investigation of Groove Shape Variation during Steel Sheave Spinning

**DOI:** 10.3390/ma11060960

**Published:** 2018-06-06

**Authors:** Chengcheng Zhu, Dean Meng, Shengdun Zhao, Shuaipeng Li

**Affiliations:** 1 School of Mechanical Engineering, Xi’an Jiaotong University, Xi’an 710049, China; sdzhao@mail.xjtu.edu.cn (S.Z.); yicunguangyin@foxmail.com (S.L.); 2 Department of Mechanical Engineering, National University of Singapore, Singapore 119260, Singapore; 3 School of Mechanical Engineering, Northwest Polytechnical University, Xi’an 710072, China; 4 School of Engineering Technology, Purdue University, 401 N. Grant Street, West Lafayette, IN 47906, USA

**Keywords:** groove shape, steel sheaves, counter-roller spinning

## Abstract

Large sheaves, such as crosshead sheaves, are foundation parts in the heavy industry. Counter-roller spinning steel sheaves are utilized to replace traditional casting iron parts. Few studies exist on the groove shape of these spinning sheaves. Consequently, it is significant to explore the spinning groove shape variation rule and confirm the appropriate spinning parameters. Both experimental and numerical methods were utilized to study the groove shape variation of Q235 steel sheaves and their results were well matched. Spring-back phenomena were considered in this study. The groove depth was lower than the spinning depth and the last formed groove was the deepest. The former groove depth would be affected by the adjacent following spinning process. The single groove spinning result was linearly dependent on the multiple spinning groove depth. Certain equations were used to calculate the groove depth. The bottom–middle–top spinning sequence, which was better than other spinning sequences, should be used in the sheave spinning method. A sheave spinning process could be designed based on the study to obtain a fine groove shape.

## 1. Introduction

Large sheaves are used in various domains, such as crosshead sheaves for elevators and traction wheels for cranes. The counter-roller spinning method, which was developed to produce high-quality large sheaves, is of interest to many suppliers and consumers [1]. Compared to other manufacturing methods, including casting and machining for large sheaves, the counter-roller spinning method has certain apparent advantages, such as being environmentally friendly and energy- and cost-saving [2,3].

Only a few studies exist on groove shape variation during counter-roller spinning. The groove shape is significant for sheaves [4,5]. An extremely large groove would lead to the cope becoming oblate, whereas an extremely small groove cannot retain the cope at the right position. Consequently, it was necessary to study the shape variation during forming and confirm the fine parameters. Q235 steel, which is a common sheave material, was used in this study [6].

The traditional metal spinning method has been widely studied. Elknabeery et al. observed that through the decrease of the roller angle and feeding rate, the spinning quality of an aluminum cup could be improved [7]. Jia et al. explored non-axisymmetric die-less shear spinning through simulations and experiments [8]. Eamonn and John studied a method to maintain the thickness during spinning through theoretical analysis and experimentation [9]. Liang et al. studied the roller effect on the splitting spinning result with the finite element method (FEM) [10,11]. Long and Hamilton explored the thickness distribution in conventional spinning from the strain viewpoint [12]. Wong et al. discussed the way to spin the plate into a thin-walled tube [13]. Peter and David studied the flow-forming method for internally geared wheels [14]. Kawai et al. explored the shear spinning method for hemispherical shells and discovered that the common sine law was not appropriate for this condition [15]. Therefore, it could be observed that the main research methods for metal spinning were numerical simulations and experiments, which were also utilized in this study.

In order to explore the spinning shape to provide the design foundation for large sheave manufacturing, certain items should be studied. Firstly, it was required to affirm the groove shape subsequently to spinning. Next, it was necessary to explore the groove shape variation rule during spinning and discover the relationship between the groove shape and the spinning parameters. Finally, the appropriate spinning procedure for large sheaves should be ascertained.

Based on the numerical and experimental methods, the groove shapes of the Q235 steel sheave was studied. The numerical model was established with ABAQUS software (Version 6.10, Johnston, RI, USA), which was satisfactory for the metal forming simulation [16,17]. The numerical results matched the experiments well. The groove depths differed and the last groove formed was the deepest. The former spun groove depths were affected by the following groove spinning. The grove depth of single-groove spinning was linearly dependent on the groove depth of multiple-groove spinning. Certain equations were used to calculate the groove depth. Through comparisons of the groove shape, thickness reduction, and other characteristics of the spinning sheaves with other methods, the bottom–middle–top spinning sequence product was judged to have suitable technological properties and the sequence was appropriate for sheave spinning.

## 2. Numerical and Experimental Models

### 2.1. Research Methods

**Figure 1:**
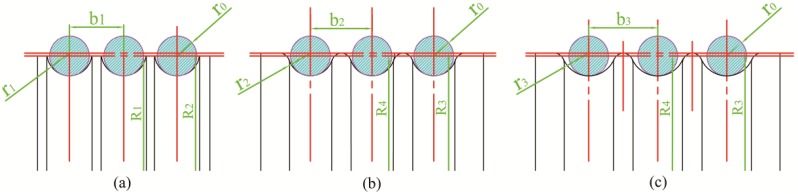
Groove shapes. (**a**) Typical groove shape for cast parts; (**b**) ideal groove shape for spinning sheaves; (**c**) general groove shape.

The groove shapes for large sheaves are presented in Figure 1. The typical groove shape for cast sheaves is a semicircle and the height center of the groove is higher than the sheave surface. The ideal spinning groove shape is presented in Figure 1b. Compared to Figure 1a, the grooves become deeper and the fillets become larger. Figure 1c presents the groove shapes that are utilized in this study. The groove size is slightly larger than the ideal situation in Figure 1b. It could be regarded as a general groove shape and considered to represent the semicircle and V-semicircle mixture types.

During spinning, all grooves were formed one by one. In a general bottom to top spinning procedure, all grooves are formed well with rectangular shapes. Consequently, the groove depth could be visualized. Therefore, the depth value was the core research object in this paper. The common groove size of 5.2 mm was selected as the basic spinning depth.

The groove depth in this study was the average value of four points measurement data acquired from the circumferential direction of the sheave. This was used to avoid the measurement errors.

Three additional feeding depths were designed to explore the relationship between the spinning parameters and the groove depth. The three feeding depths were 2 mm, 4 mm, and 6 mm. The single-groove sheave and the three-groove sheave were studied. The single-groove spinning could clearly show the spring-back effect on the groove depth, while the three-groove sheave was utilized to study the groove depths of the multiple-groove sheaves.

The relationship between the forming sequence and the groove shape was studied through the numerical method. Three typical spinning sequences were considered, the bottom to top, the top to bottom, and the bottom to top to middle. The thickness, stress, and strain of the products were used to explore the spinning sequence character.

### 2.2. Numerical Model

Two numerical models were built with ABAQUS. The three-dimensional (3D) simulation for the general study and the two-dimensional (2D) model for the spring-back study were produced. All geometric models were built in CAD software and loaded into ABAQUS. The blank was a Q235 tube 1000 mm in diameter and 1 mm in thickness. The radius of the circular bead of roller was 5.2 mm. This study is about the cold deformation at room temperature.

Both 3D and 2D models assumed a common continuous medium for the metal forming study. To save time, the mass scaling method was used in the dynamic explicit process and the scaling factor was 1000. The elastic–plastic material model was based on the Mises yield criterion, which is appropriate for metal forming. The coulomb friction method was used and the factor was set to 0.1 as the common friction factor of the spinning method. Since all rollers and mandrels were made of die steel with high hardness, they could keep a good shape during the spinning process. Therefore, the tube blank was set as the deformable part and other parts were set as discrete rigid parts. By this method, the model size could be effectively decreased.

**Figure 2:**
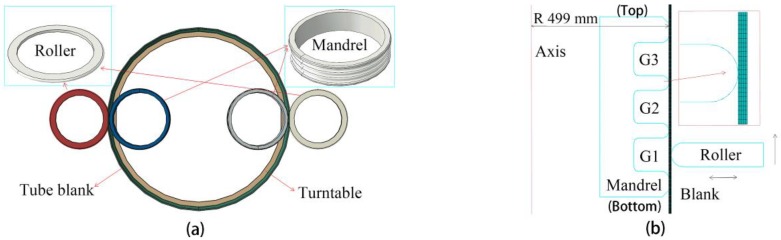
Numerical model for sheave counter-roller spinning. (**a**) 3D model; (**b**) 2D model.

The 3D model is presented in Figure 2a. During the counter-roller spinning, the tube blank was rotated with the turntable. All rollers moved in the axial and radial direction of the blank and formed all grooves one by one. The mandrels kept their position during the spinning process. Since the tube diameter was larger than its thickness, the shell element was utilized to mesh the tube blank. The continuum shell element considering the wall thickness variation was a widely used element for sheet forming. The continuum shell element SC8R was used for the tube blank. The element size should be small enough to obtain accurate results. Nine integration points were set in the radial section. The element size was 2 mm in the axial direction and 12 mm in the circumference direction. The other parts in the model were meshed with the R3D4 element with a similar element size. The rotation speed of the blank was 40 r/min. The feed speed of the outer rollers were 1 mm/s. Also, the general groove forming sequence was from the tube bottom to the top.

In ideal circumstances, the groove depth should be the same as the spinning depth, 5.2 mm. Since this was a typical sheet forming method, the spring-back phenomenon could apparently change the product shape [18,19]. Consequently, the spring-back of the spinning sheaves should be studied.

Considering that the sheave was too large to obtain a nice spring-back result in a 3D numerical simulation, the 2D model was selected to research the spring-back. As is well known, the metal spinning method is executed at the plain strain condition [20]. Also, the 2D axial symmetry model has been widely used to study the spinning and the spring-back phenomenon [21]. The 2D model is presented in Figure 2b.

In the 2D model, the element size of the blank was 1 mm. The axisymmetric element CAX4R was used. Nine layers elements were set in the radial direction to improve the simulation accuracy. The roller and mandrel were meshed with the RAX2 element with a 1 mm size. The dynamic explicit method was utilized for the spinning process simulation and the standard method was selected to calculate the spring-back value.

The material properties of the Q235 steel were obtained through the material performance measurements presented in Table 1. The Q235 steel flow stress curve from the tensile experiment is shown as in Equation (1). The Hollomon equation was utilized to fit the flow stress curve:

(1) $$S=969.1e^{0.7973}+278.6,$$

where S is the flow stress and e is the strain.

### 2.3. Experiments

**Figure 3:**
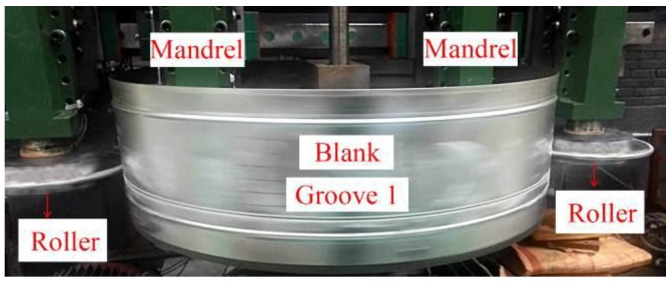
Experiment during Groove 1 spinning.

The experiments were conducted with a computer-controlled counter-roller spinning machine. The experimental model is presented in Figure 3. The blank was a Q235 tube 1000 mm in diameter and 1 mm in thickness. The same parameters were used in the numerical model. Two small preformed grooves existed near the sides of the tube blank to improve the tube rigidity. The tube blank was fixed on the main shaft of the spinning machine and rotated at a speed of 40 r/min. Grease was used as the lubricant during experimentation. Also, the surfaces of the rollers, the mandrels, and the tube blank should be wiped prior to the grease smearing. The spinning parameters were also the same in the numerical model.

## 3. Results and Discussion

### 3.1. General Results

**Figure 4:**
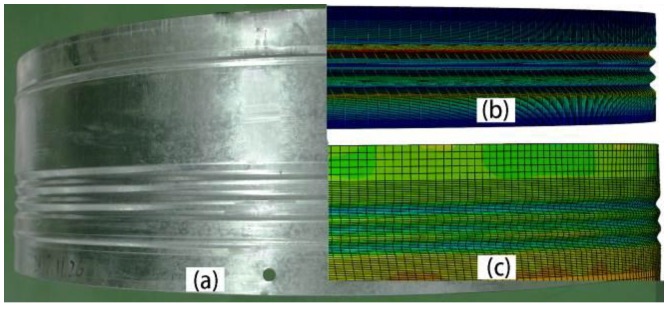
Results of experimental and numerical simulation. (**a**) Experimental result; (**b**) 2D numerical simulation; (**c**) 3D numerical simulation.

The results of numerical simulations and experiments are presented in Figure 4, while the simulation results matched the experimental results well. The stress and strain in both two numerical simulations were at a low level. No visible cracks or other defects existed on all products. As significant parameters of the spinning process, the spinning force and the product wall thickness were studied to compare the experimental and numerical results. The spinning force and wall thickness information were all at the 5.2 mm spinning depth condition. The forming force during the spinning process of the experimental and numerical simulation (3D) was 8.28 kN and 8.71 kN, respectively. The simulation force was larger than the experimental force by about 5.2%. The average wall thickness in all experimental and numerical results was about 0.99 mm. Therefore, it was feasible to use the numerical method to study the counter-roller spinning.

**Figure 5:**
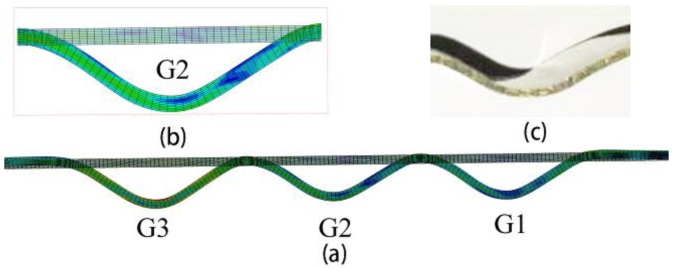
Final groove shapes. (**a**) Final result and origin blank; (**b**) enlarged view of G2; (**c**) groove section of experimental result.

The final grooves are presented in Figure 5. Comparing the groove section between the numerical and experimental results shown in Figure 5b,c, they had very similar shape and depth. They all had a smooth surface without any defects. The numerical and experimental finished parts had s similar wall thickness and groove depth distribution. The depths of the G1 and G2 were similar. The G3 depth was slightly greater than the other two depths. The majority groove tops were higher than at the initial position.

**Figure 6:**
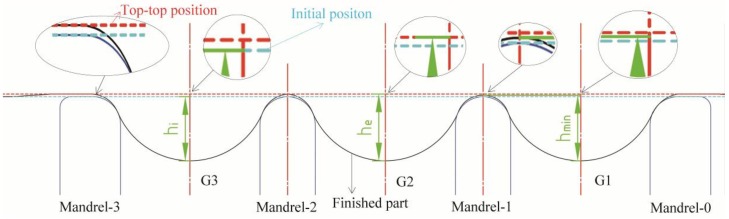
Groove depth measurement methods.

The depth measurement method is presented in Figure 6. The finished part’s thickness was nearly similar to the blank. Consequently, the thickness of the blank was ignored. Depending on the different reference positions of depth, three depth measurements were conducted. The first was the direct testing of the displacement of the groove bottom at the ideal groove depth, $h_{i}$. This was convenient to obtain in the numerical simulation, but hard in the experiments. The second method was the distance measurement among the groove bottom and its adjacent tops. The minimum depth among the bottom and its adjacent tops was the valid groove depth $h_{min}$. This was also difficult to find during experimentation. Therefore, the average depth between the bottom and adjacent top-top line could be measured as $h_{ave}$. A shortcoming of this method was that each groove depth had a different reference point, which was not convenient to compare to every other point. The third method used a certain reference line to measure all groove depths $h_{e}$. The top-top line between Mandrel-0 and Mandrel-1 was selected as the reference line for both the experiments and the numerical simulation. It was convenient to obtain and compare the depths between each groove. This was the main measurement method used in this study. The depth was measured with a precision Vernier caliper during experimentation. The top line was obtained through the setting of a 0.5 mm standard plug gauge on the surface of the spinning sheave.

**Figure 7:**
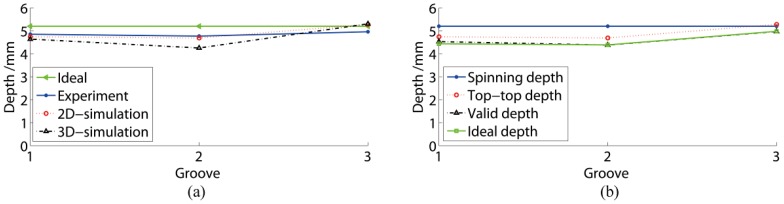
Comparisons of groove depths. (**a**) Comparison between experiments and numerical simulation; (**b**) comparison of three measurement methods.

The depths of the three grooves are presented in Figure 7a. In an ideal situation without spring-back phenomena, all three groove depths were 5.2 mm. The depths of Grooves 1, 2, and 3 in the experiments were 4.85 mm, 4.77 mm, and 4.96 mm, respectively. The depths of Grooves 1, 2, and 3 in 2D simulation were 4.74 mm, 4.69 mm, and 5.28 mm, respectively. Through the experimental result and 2D simulation comparisons, the depths of G1 and G2 matched well, while the depth of G3 was slightly higher with a deviation of 6.45%. The 3D simulation without spring-back had an apparently higher deviation than the 2D simulation, especially for the G3 depth.

The results of the three measurements in the 2D simulation are presented in Figure 7b. The valid and ideal depths were similar, nearly 0.25 mm less deep than the top-top depth. All these depth curves had the same variation rule. Consequently, it was efficient to use the top-top depth to study the groove shape distribution.

The spring-back of G1 was approximately 0.40 mm and 7.69% of the ideal depth. The spinning affected the groove depth. The latest formed groove was the deepest. G3 was nearly 0.16 mm deeper than the G1 and G2.

Actually, the dynamic explicit simulation could also demonstrate the spring-back phenomenon, but it required significant running time to obtain the final result. Considering the time consumption and data accuracy, the 2D simulation was better than the 3D simulation for the groove shape study. The 2D numerical method could produce results that exactly matched the experiments. Therefore, the 2D simulation was regarded as the major numerical method.

### 3.2. Depth Variation during Spinning

**Figure 8:**
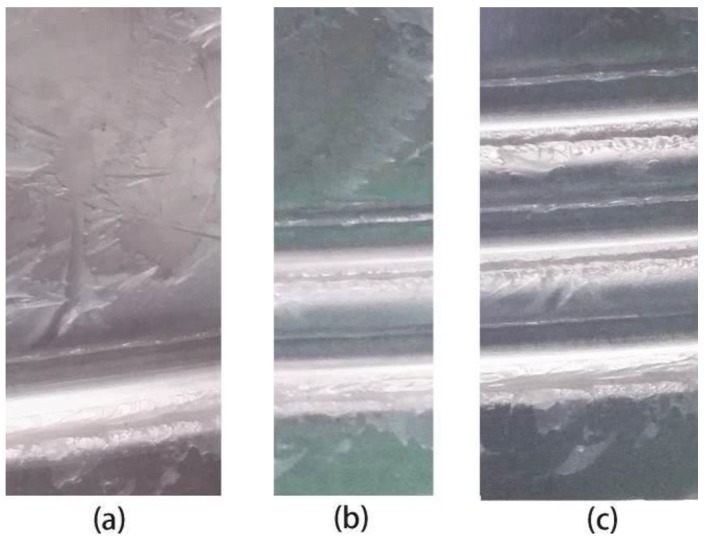
Results following each spinning step. (**a**) First step; (**b**) second step; (**c**) third step.

The experimental results following each groove spinning step are presented in Figure 8. All grooves were formed with good shapes. The depth of each groove was measured subsequent to every spinning step. The groove depths during the entire spinning varied.

**Figure 9:**
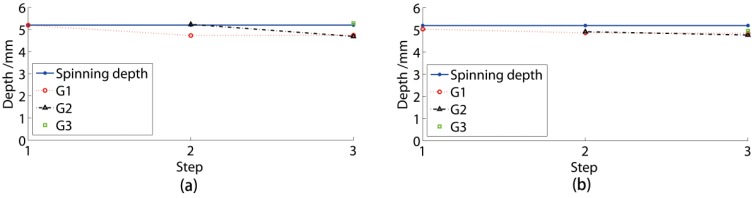
Groove depth during spinning. (**a**) Numerical; (**b**) experimental.

The results of each numerical simulation process step were the same as in the experiment. The numerical groove depth during spinning is presented in Figure 9a. The last finished groove of each step had a similar depth to the ideal spinning depth of 5.2 mm. The former groove depth became lower during the spinning that followed. The groove depth of G1 was retained in the G3 spinning. Also, the depths of G1 and G2 were similar following the G3 spinning. The final top-top depth of the groove spinning was approximately 4.7 mm and the efficient depth was approximately 4.5 mm.

The experimental results are presented in Figure 9b. The groove depth variation rules were similar in both numerical simulations and experiments. Each groove in the experiment had a slightly lower initial depth value compared to the numerical simulation, but the experimental grooves had higher final depths than the numerical grooves. The initial and final depths in the experiments were approximately 4.96 mm and 4.8 mm, respectively. The average deviation of the experimental and numerical result was below 4.8%. Therefore, it was efficient to use the numerical simulation to study the groove depth.

**Figure 10:**
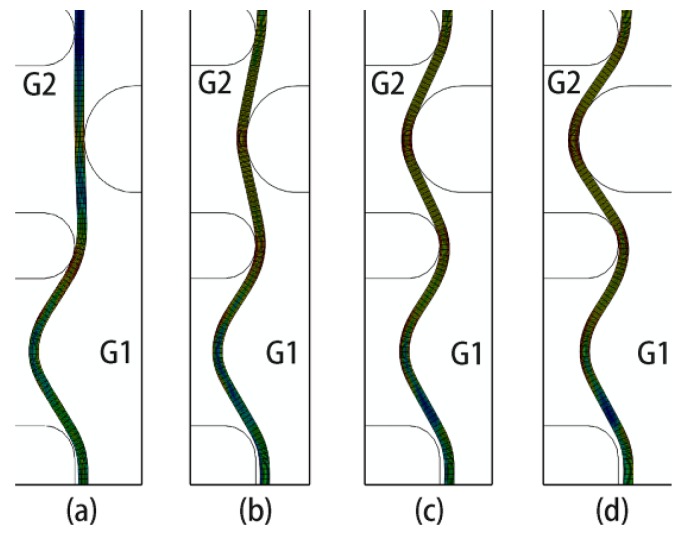
G1 deformation during G2 spinning. (**a**) 0 mm depth; (**b**) 2 mm depth; (**c**) 4 mm depth; (**d**) 5.2 mm depth.

**Figure 11:**
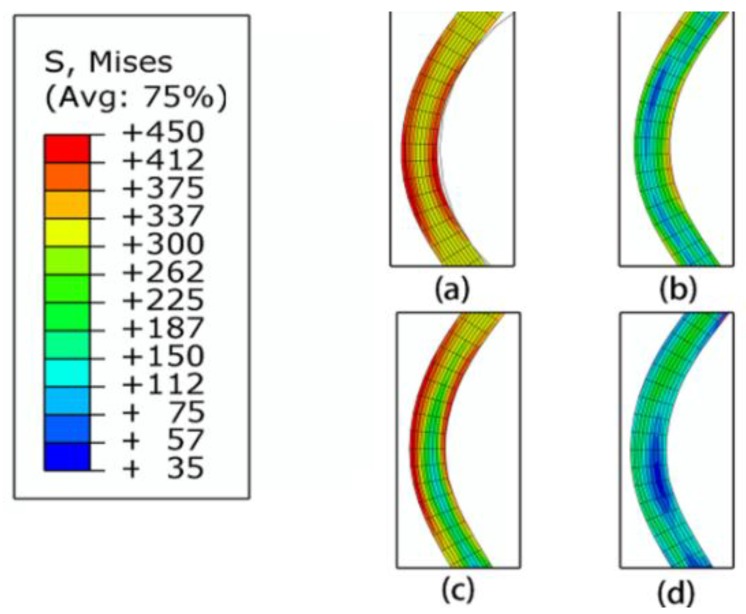
Stress variation of G1 bottom during spinning. (**a**) G1 spinning; (**b**) following G1 spinning; (**c**) G2 spinning; (**d**) following G2 spinning.

Based on the numerical simulation, it could be observed that three main reasons existed for the former groove depth’s decrease. The first reason was the material flow in the axial direction and the second reason was the warping of the blank. The material flow and blank warping could be observed in Figure 10. The element at the G1 area moved slightly to G2 during the G2 spinning, while the area near G1 produced warping, leading to the groove elements’ movement to the mandrel exterior. The stress variation could clearly demonstrate the deformation state during spinning. The G1 stress during spinning is presented in Figure 11. The G1 was affected by the G2 spinning as the stress increased from 247 MPa to 448 MPa. Following the G2 spinning, the G1 stress was approximately 159 MPa, which was significantly lower than the stress subsequent to the G1 spinning of 247 MPa. Consequently, the stress was released by the G2 spinning and the total deformation was lower. This was the third reason for the former groove depth decrease.

Since the groove between the forming zone and the routine could restrict both the material flow and warping from routine groove, the routine groove depth would not change during the following spinning. Consequently, the G1 depth significantly decreased during G2 spinning, but was nearly constant during G3 spinning. The final groove depth could be regarded as a random groove depth subsequent to the adjacent groove spinning.

### 3.3. Relationship between Groove and Spinning Depths

#### 3.3.1. Spring-Back of Spinning Groove

The spinning depth is the key parameter, which determines the ideal groove shape in the sheave spinning method. The spring-back is the phenomenon leading to the difference between the ideal spinning groove and the final groove.

**Figure 12:**
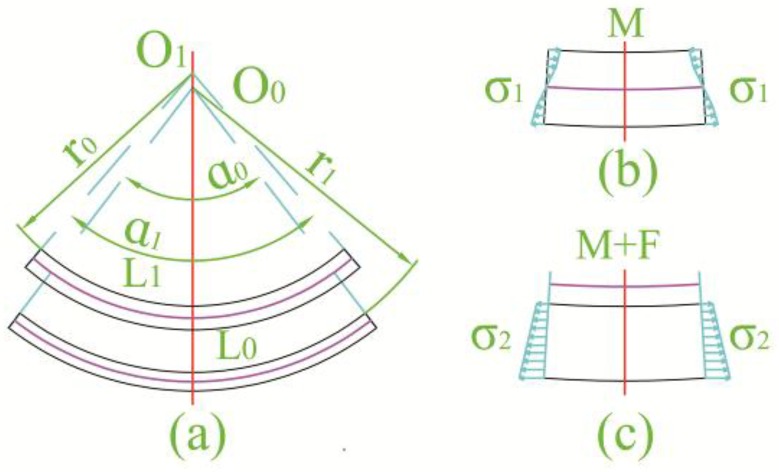
Spring-back model. (**a**) Deformation during spring-back; (**b**) stress state of general bending; (**c**) stress state of spinning groove.

The theory model of the spring-back phenomenon is shown in Figure 12. The simple spring-back model as the bending method is presented in Figure 12a,b [22,23,24]. In the general bending method, the inner torque determined the deformation of the blank. The neutral line with a constant length during forming was simply at the middle of the blank. The layer inside the neutral line had compression plastic stress and the layer outside the neutral line had tensile plastic stress. If the external force was removed, the internal stress of the blank would be redistributed to maintain the internal torque balance to lead the spring-back. The spring-back often causes radius and angle changes in the formed part. Actually, the stress state of the groove spinning could be simplified to the stretch bending in Figure 12c. The stress of the blank was tensile and the “neutral line” was out of the blank [25]. The first spring-back step of the spinning groove was the shrinkage of the blank in the circumference direction. Subsequently, the rest of the spring-back process was similar to the general bending method. The material properties, geometrical shape, and other spinning parameters affect the spring-back phenomenon. It was difficult to discover a general theoretical equation to calculate the spring-back value. Consequently, it was necessary to use both the numerical and experimental methods to study the spring-back of the spinning groove.

#### 3.3.2. Single-Groove Spinning

The single-groove sheave is a widely utilized part such as pulleys. Also, the single-groove study could directly produce the groove spinning characteristics without the effects of other grooves. Consequently, it was necessary to study the single-groove spinning method.

**Figure 13:**
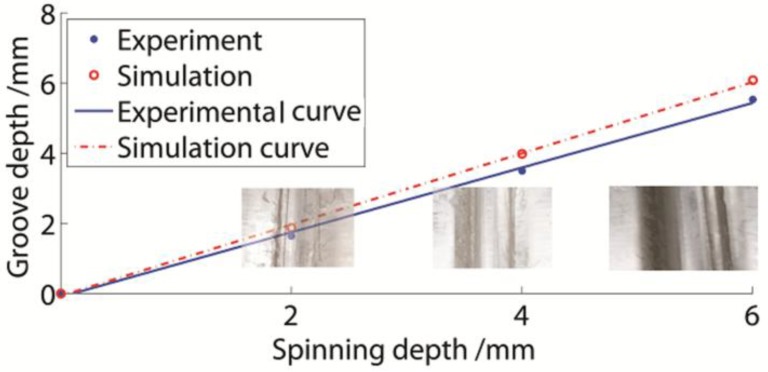
Groove depth variation of single-groove sheaves.

The relationship between the groove and spinning depths is presented in Figure 13. The groove shape in each experiment is also shown in Figure 13. The spinning depths were 2 mm, 4 mm, and 6 mm. All groove depths were lower than the spinning depths. As the spinning depth increased, the groove depth increased linearly. The numerical value was slightly higher than the experimental value.

The general spring-back empirical equation for stretch bending, which includes the similar stress states of groove spinning, often has apparent distortions in practical situations, and it is hard to make the coefficients conform [26]. Therefore, it was necessary to discover a suitable empirical equation for groove spinning. The function fitting equations based on the experimental and numerical data, which constitute a precise empirical method for a specific manufacturing process, are presented as Equations (2) and (3):

(2) $$h_{exp}=0.922H-0.101,$$

(3) $$h_{num}=1.016H-0.07048,$$

where $h_{exp}$ is the experimental groove depth, H is the spinning depth, and $h_{num}$ is the numerical groove depth. The R-square of both equations exceeded 0.998. Consequently, it was accurate to describe the groove depth variation rules. It could be observed that the numerical groove depth was simply 1.102 times the experimental result.

The spring-back phenomenon of the spinning groove was similar to the stretch bending process. Therefore, the assumption of the spring-back was accurate to analyze the spring-back phenomenon of the spinning groove. Moreover, certain methods for the spring-back decrease of the spinning depth could also be used in groove spinning. The single-groove depth could be obtained through these equations.

#### 3.3.3. Three-Groove Spinning

**Figure 14:**
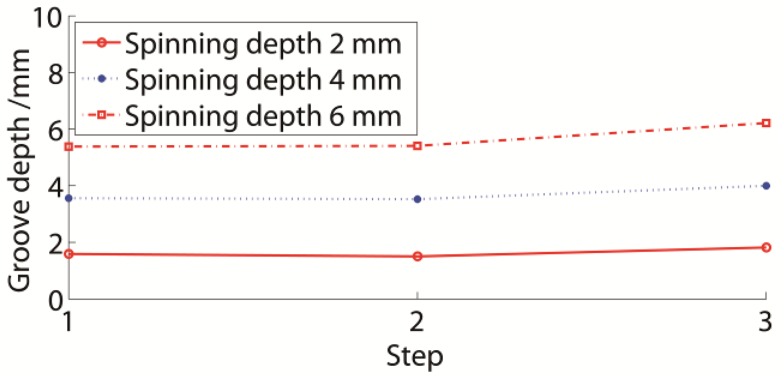
Groove depth variation of three-groove sheave during spinning.

The groove depth of a multiple-groove sheave is affected by both the common spring-back phenomenon and the following spinning. The three-groove sheave was selected as an example of multiple-groove sheaves. If the 2 mm, 4 mm, and 6 mm spinning depths were used to form the three-groove sheaves, the numerical results would be as presented in Figure 14. These curves were similar to the curves in Figure 7, being almost paralleled. A larger spinning depth and groove depth could be observed. The last finished groove was always larger than the former grooves, while the depths of previously formed grooves were nearly similar in the following spinning process. The final groove depths under the 2 mm, 4 mm, and 6 mm spinning depth conditions were 1.54 mm, 3.54 mm, and 5.39 mm, respectively.

**Figure 15:**
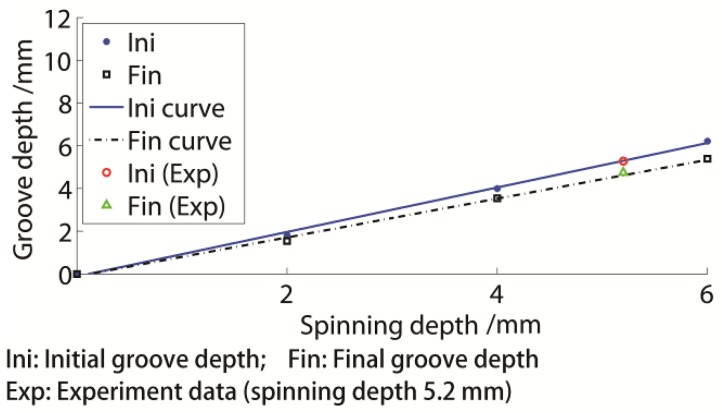
Groove depth variation of three-groove sheave.

The final and initial groove depths under different conditions are presented in Figure 15. All initial and final groove and spinning depths were linearly dependent. Therefore, the two fitting equations for the initial and final groove depths are presented as in Equations (4) and (5):

(4) $$h_{i}=1.041H-0.1166,$$

(5) $$h_{fin}=0.9085H-0.108,$$

where $h_{ini}$ is the initial groove depth and $h_{fin}$ is the final groove depth. The R-squares of both equations exceeded 0.997. The final groove depth was approximately 87.27% of the initial groove depth.

If the experimental result from the 5.2 mm spinning depth situation was inserted into Figure 15, the fitting equation would match the result well. Consequently, it was efficient to use the equation to design the spinning depth, to obtain the target groove.

Through a comparison of the results of single-groove and three-groove spinning, their groove depth change rules were similar, as linearly dependent on the spinning depth. Since the final multiple-groove depth variation was the superposition of the common spring-back of the single groove and affected the following groove spinning, the following groove spinning effect was also linearly dependent on the spinning depth.

The final groove depth of the single-groove spinning was slightly higher than that of the three-groove spinning. Based on the four previous equations, $h_{ini}$ was approximately 1.1291 times the $h_{exp}$, while $h_{fin}$ was approximately 0.9854 times the $h_{exp}$. Depending on the study, both the spinning depth and the single-groove depth could be used to calculate the groove depth of the three-groove spinning. The equations were also efficient for other multiple-groove spinning processes.

### 3.4. Effect of Groove Spinning Sequence

The three-groove sheave had a fixed side (bottom side), as presented in Figure 3. Considering the symmetry and operation of the spinning model, two major and four additional spinning sequences existed. The two major spinning sequences were bottom to top (G1-G2-G3 or B-T-M) and top to bottom (G3-G2-G1 or T-M-B). The four additional spinning sequences, which should miss certain grooves during spinning, resulted in groove shape inconsistency. Therefore, only one additional spinning sequence, bottom-top-middle (G1-G3-G2 or B-T-M), was studied as an example. The spinning depth was 5.2 mm.

**Figure 16:**
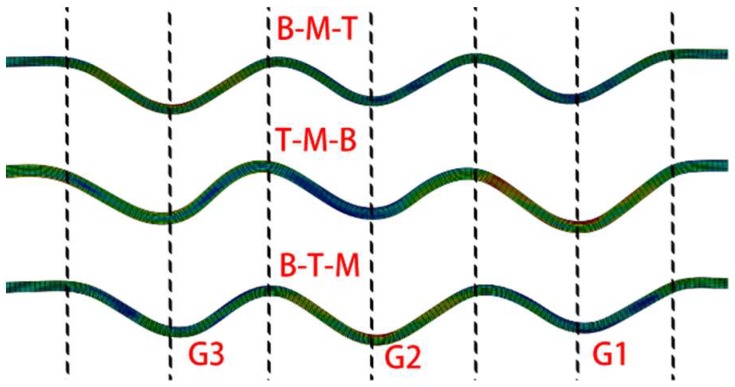
Results of three spinning sequences.

The results of the three spinning methods are presented in Figure 16. The B-M-T experimental result is shown in Figure 8. Considering that the B-M-T experimental and numerical results matched well, the numerical method is efficient in this spinning study. Several differences were observed between the results. The B-M-T method could produce the best groove shape during these spinning methods. All groove shapes in the T-M-B and B-T-M had a certain amount of distortion at the bottom, near the following formed groove. Also, in the B-M-T result, all groove bottoms maintained almost their initial position at the middle of each groove. Therefore, it was efficient to use the groove depth to study the B-M-T spinning method. Moreover, the groove depth in the B-M-T method was more uniform and higher than in the other methods.

**Figure 17:**
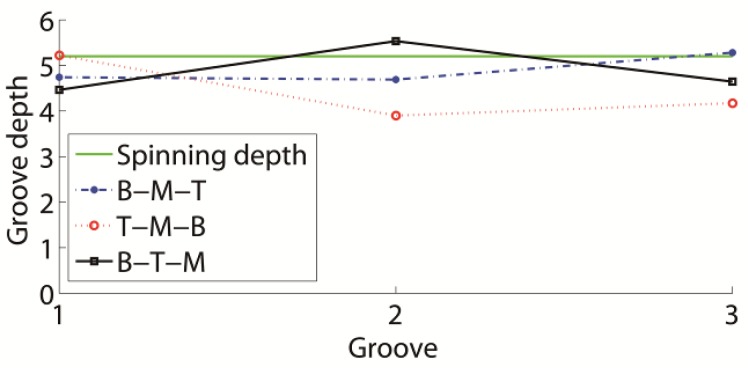
Effect of spinning sequence on groove depth.

The groove depth of each spinning result differed. The top–top groove depths of these spinning sequences are presented in Figure 17. The final groove depths of B-M-T, T-M-B, and B-T-M were 4.8 mm, 3.9 mm, and 4.5 mm, respectively. The final groove depth of the B-M-T was higher compared to the other two methods. The higher groove depth signified that the spinning method had better capacity to maintain the spinning depth. Actually, if the efficient groove depths were compared, the B-M-T method produced better values compared to the other methods. The efficient groove depths of B-M-T, T-M-B, and B-T-M were 4.5 mm, 3.34 mm, and 3.83 mm, respectively.

The stress, strain, and thickness results of these spinning processes are presented in Table 2. The sheet forming processing should control the blank thickness to ensure the product quality. Although in all results the thicknesses were well maintained, the T-M-B method had the highest thickness reduction compared to the other methods, because the T-M-B spinning produced a higher amount of local forming than the other methods.

In all spinning results, the maximum stress and strain were produced at the mandrel upper surface fillets. The stress and strain of the B-M-T were slightly higher than in the other two methods, but at a low level compared to the material properties. Also, both the stress and strain during spinning were tensile. Consequently, all these spinning processes could spin the grooves almost without cracking, wrinkles, or other defects.

Considering all the aforementioned conditions, in the B-M-T spinning sequence with the best groove shape and acceptable strain, stress, the thickness result was satisfied for the sheave spinning process. The B-M-T sequence should be considered the first choice for sheave spinning.

## 4. Conclusions

The groove shapes of the spinning sheaves were studied with numerical and experimental methods. The general spinning groove shape characters, the groove depth distribution, the groove depth variation during spinning, the groove depth calculation and the spinning sequence were discussed.

1. The grooves of all 3D/ 2D numerical simulations and experiments were well formed. Also, the 2D numerical simulation, when the spring-back phenomenon was considered, matched the experimental results well. Therefore, the numerical method was efficient for the groove spinning study.
2. The last formed groove was always the deepest of all grooves, while the former spun grooves had similar depths. When the spinning depth was 5.2 mm, the top–top groove depths of the last and former grooves were approximately 5.28 mm and 4.70 mm, respectively. Moreover, their valid groove depths were approximately 0.25 mm shallower than the top–top groove.
3. The groove depth changed during spinning. The former groove depth was almost constant subsequent to the following spinning. The depth of Groove 1 was highly decreased during the second groove spinning, and changed slightly during the third groove spinning. The Groove 2 depth became lower, with a value equal to the final Groove 1 depth, following the third groove spinning. Therefore, the final groove depth of the spinning sheave could be regarded as the groove depth following the next groove spinning.
4. The groove depth was nearly linearly dependent on the spinning depth. The groove depth of the single-groove spinning was slightly higher than the three-groove spinning. In addition, the single-groove depth could be used to calculate the multiple grooves’ depths. Equations for the relationship between the groove depth and the spinning depth as well as between the single and multiple groove depths were obtained. The groove processing could be designed with these equations.
5. The groove spinning sequences had an apparent effect on the spinning process. The B-M-T sequence was the most suitable for the sheave spinning. The B-M-T results were better groove shapes and higher groove depths than the other results. Also, the B-M-T result was a well-retained wall thickness, including similar stress and strain values to the other results. Therefore, B-M-T should be selected in the sheave spinning process.

## Figures and Tables

**Table 1:** Material properties of Q235 steel.

Density/kg/m^3^	Young’s Modulus/GPa	Poisson’s Ratio	Yield-Strength/MPa	Ultimate-Strength/MPa
7219	200	0.3	310	607

**Table 2:** Stress, strain, and thickness results of three spinning processes.

Items	B-M-T	T-M-B	B-T-M
Maximum-stress/MPa	464.8	458.6	458.0
Maximum-strain	0.1246	0.1177	0.1172
Minimum-thickness/mm	0.9878	0.9752	0.9890
